# Development and validation of a novel cellular senescence-related prognostic signature for predicting the survival and immune landscape in hepatocellular carcinoma

**DOI:** 10.3389/fgene.2022.949110

**Published:** 2022-09-06

**Authors:** Rui Sun, Xu Wang, Jiajie Chen, Da Teng, Shixin Chan, Xucan Tu, Zhenglin Wang, Xiaomin Zuo, Xiang Wei, Li Lin, Qing Zhang, Xiaomin Zhang, Kechao Tang, Huabing Zhang, Wei Chen

**Affiliations:** ^1^ Department of General Surgery, First Affiliated Hospital of Anhui Medical University, Hefei, China; ^2^ Department of Dermatology, First Affiliated Hospital of Anhui Medical University, Hefei, China; ^3^ Department of Hepatopancreatobiliary Surgery, Affiliated Chuzhou Hospital of Anhui Medical University, First People’s Hospital of Chuzhou, Chuzhou, China; ^4^ Department of Biochemistry and Molecular Biology, Metabolic Disease Research Center, School of Basic Medicine, Anhui Medical University, Hefei, China; ^5^ Affiliated Chuzhou Hospital of Anhui Medical University, First People’s Hospital of Chuzhou, Chuzhou, China

**Keywords:** hepatocellular carcinoma, lncRNA, senescence, prognosis, immune landscape

## Abstract

**Background:** Cellular senescence is a typical irreversible form of life stagnation, and recent studies have suggested that long non-coding ribonucleic acids (lncRNA) regulate the occurrence and development of various tumors. In the present study, we attempted to construct a novel signature for predicting the survival of patients with hepatocellular carcinoma (HCC) and the associated immune landscape based on senescence-related (sr) lncRNAs.

**Method:** Expression profiles of srlncRNAs in 424 patients with HCC were retrieved from The Cancer Genome Atlas database. Lasso and Cox regression analyses were performed to identify differentially expressed lncRNAs related to senescence. The prediction efficiency of the signature was checked using a receiver operating characteristic (ROC) curve, Kaplan–Meier analysis, Cox regression analyses, nomogram, and calibration. The risk groups of the gene set enrichment analysis, immune analysis, and prediction of the half-maximal inhibitory concentration (IC50) were also analyzed. Quantitative real-time polymerase chain reaction (qPCR) was used to confirm the levels of *AC026412.3*, *AL451069.3*, and *AL031985.3* in normal hepatic and HCC cell lines.

**Results:** We identified 3 srlncRNAs (*AC026412.3*, *AL451069.3*, and *AL031985.3*) and constructed a new risk model. The results of the ROC curve and Kaplan–Meier analysis suggested that it was concordant with the prediction. Furthermore, a nomogram model was constructed to accurately predict patient prognosis. The risk score also correlated with immune cell infiltration status, immune checkpoint expression, and chemosensitivity. The results of qPCR revealed that AC026412.3 and AL451069.3 were significantly upregulated in hepatoma cell lines.

**Conclusion:** The novel srlncRNA (*AC026412.3*, *AL451069.3*, and *AL031985.3*) signatures may provide insights into new therapies and prognosis predictions for patients with HCC.

## 1 Introduction

Cancer is one of the leading causes of death worldwide (Bray et al., 2021). More than 19 million new cancer cases and nearly 10 million cancer-related deaths have been reported in 2020, including over 900,000 new liver cancer cases and 800,000 related deaths (Sung et al., 2021). Liver cancer has the seventh highest incidence among all cancer types and the third highest mortality rate. Hepatocellular carcinoma (HCC) is the most common type of liver cancer. East Asia and Africa have the highest incidence rates of HCC, and its incidence and mortality rates are still increasing in Europe and other parts of the world (Llovet et al., 2021; Sung et al., 2021). Owing to the progress of surgery and chemotherapy, the prognosis of patients with HCC has greatly improved, and the progress of tumor immunotherapy and the use of immune checkpoint inhibitors have also improved the treatment strategies for HCC treatment (Bagchi et al., 2021). However, more efficient molecular biomarkers for the early diagnosis of HCC are crucial for improving the clinical outcomes of patients with HCC.

Cellular senescence is a typical irreversible form of life stagnation that helps inactivate and eliminate diseased, dysfunctional, and other unnecessary cells. It is usually induced by various conditions, such as microenvironmental stress, damage to organelles and cellular infrastructure, and an imbalance of cellular signal networks. However, all these conditions are related to the increase in senescent cell abundance in various organs observed during the aging process. It is considered to be one of the basic hallmarks of cancer (Hanahan, 2022).

Long non-coding ribonucleic acids (lncRNAs) are composed of >200 nucleotides that cannot be translated into functional proteins (Iyer et al., 2015). In the human genome, there are more than 100,000 identified lncRNAs, many of which have been characterized (Heery et al., 2017). lncRNAs are usually the main regulators of gene expressions and functions through post-transcriptional, transcriptional, and epigenetic regulation (Castro-Oropeza et al., 2018). Previous studies have shown that lncRNAs can influence the immune microenvironment; therefore, they may have a role in the occurrence and development of malignancy (Atianand et al., 2017). The HOX transcript antisense RNA was found to be upregulated in colon tumor tissues and correlated with the tumor stage, invasion, metastasis, and survival time of patients (Luo et al., 2016; Tatangelo et al., 2018; Wei et al., 2020); it is also associated with cancer growth and metastasis (Wei et al., 2020). Zhao et al. (2019) reported that the knockdown of lncRNA myocardial infarction–associated transcript significantly promoted cellular senescence and inhibited HCC progression. Montes et al. (2021) identified MIR31HG as a potential therapeutic target in the treatment of senescence-related pathologies. The effects of senescence-related (sr) lncRNAs on malignant tumors have not been well studied; therefore, obtaining more knowledge on srlncRNAs will help us better understand their roles in cancer therapy.

In recent years, many studies have developed signatures for predicting cancer prognosis based on coding genes or non-coding RNAs. Kandimalla et al. (2020) identified a signature for predicting the survival in pancreatic ductal adenocarcinoma. Chen et al. (2021) constructed a prognosis index for head and neck tumors using immune-related genes. Zhou et al. (2021) identified an immune-related lncRNA signature to predict the survival and the immune landscape in patients with HCC. However, only a few studies have focused on signature development using srlncRNAs.

This study aimed to determine the value of srlncRNAs in predicting the prognosis and immune landscape of HCC, thus contributing to this growing area of research. Our findings may help improve our understanding of the role of cellular senescence in HCC and lead to progress in treatment strategies.

## 2 Materials and methods

### 2.1 Data collection

RNA-seq expression data derived from patients with HCC, including 374 tumors and 50 non-cancerous samples, were collected from The Cancer Genome Atlas (TCGA) database with the TCGA-Assembler. Based on the patients’ IDs, the clinical data of the patients were compared to their transcriptome data, which were screened using the following inclusion criteria: [1] histological diagnosis of HCC, [2] available expression profiles, and [3] a minimum overall survival of 30 days (Song et al., 2021). The data satisfying the inclusion criteria were extracted from the TCGA dataset (344 patients) for subsequent analysis, and 279 senescence-related genes (explained in Supplementary Table S1) were retrieved from the literature search and the CellAge public database.

### 2.2 Identification of senescence-related long non-coding ribonucleic acids

The association between lncRNAs and senescence-related genes (SRGs) was assessed using Pearson’s correlations to identify srlncRNAs. Using the Bioconductor limma package in R software (version 4.1.3), HCC and non-neoplastic samples were compared, and differentially expressed lncRNAs (DElncRNAs) were defined with the following criteria: |log2 (fold change, FC) | >1 and false discovery rate < 0.05 (Ritchie et al., 2015). A total of 279 senescence-related genes and those of DElncRNAs were identified by using the correlation analysis. Hence, 422 srlncRNAs were selected based on the following criteria: Pearson’s correlation coefficients > 0.5 and *p* < 0.001.

### 2.3 Construction of the senescence-related lncRNA prognostic model

First, we randomly divided the patients from the entire sample (*n* = 342) into training or testing sets at a rate of 1:1. Second, srlncRNAs (related to survival) in the training set were screened using univariate Cox (uni-Cox) regression (*p* < 0.05). Third, least absolute shrinkage and selection operator (LASSO) and multivariate Cox (multi-Cox) regression analyses were used for further filtering. Finally, a prognostic model for srlncRNAs was established in HCC. We calculated the risk score for HCC as follows: risk score=∑n k=1expression (lncRNAk) × coefficient (lncRNAk) (Hong et al., 2020). Using the median value, we divided the cases into two groups: high and low. Moreover, testing sets were employed for signature validation. The signature was associated with clinical variables using the chi-square test. The Wilcoxon signed-rank test was performed to identify differences in the risk scores between the groups for clinical characteristics. Furthermore, the R package “rms” was used to build a nomogram model that connected the signature risk score and clinical factors, and calibration curves were used to assess the model (Iasonos et al., 2008).

### 2.4 Gene set enrichment analysis

Using the curated gene set (kegg.v7.4.symbols.gmt), broad GSEA v.4.2.3 was applied to detect high- and low-risk group-correlation pathways with the criteria: NOM *p* < 0.05 and | NES |> 1 (Subramanian et al., 2005).

### 2.5 Infiltrating immune cell analysis

The immune infiltration statuses calculated in the datasets (XCELL, TIMER, QUANTISEQ, MCPcounter, EPIC, CIBERSORT, and CIBERSORT) and the infiltration estimation downloaded in TIMER2.0 (http://timer.cistrome.org/) were used to analyze the diﬀerences in immune infiltrating cell content using the Wilcoxon signed-rank test. Using the profile of infiltration estimation for HCC on that website, a bubble chart was created showing the differences in immune infiltrating cell content using the Wilcoxon signed-rank test and the following R packages—“limma”, “scales”, “ggplot2”, and “ggtext” (Bagchi et al., 2021).

### 2.6 The investigation of the immune checkpoints and immune-related gene prognostic index

The “ggpubr” R package was used to compare the expression of immune checkpoint-related genes between the two groups. The multi-Cox regression analysis was used to construct an IRGPI model to validate the impact of the prognostic model on immunotherapy.

### 2.7 The sensitivity of different subgroups to chemotherapeutic agents

We used the half-maximal inhibitory concentration (IC50) to evaluate the therapeutic effects of common chemotherapeutic drugs (paclitaxel, doxorubicin, bexarotene, bicalutamide, imatinib, and tipifarnib) using the R package “pRRophetic” with data collected from the Genomics of Drug Sensitivity in Cancer.

### 2.8 RNA isolation and quantitative real-time PCR

Total RNA was extracted from hepatoma cell lines (Huh7, HepG2, and Hep3B) and a normal hepatic cell line (LO2) using TRIzol reagent (Life, United States). NanoDrop 2000 (Thermo Scientific, America) was used to measure RNA purity and content. Complementary DNAs were synthesized using a RevertAid RT kit (Thermo, United States), and qPCR was performed on a Bio-Rad CFX system using qPCR Master mix (Universal, China). The sequences of the primers used for qPCR were as follows: *AC026412.3*, forward: 5′-TGT​GAG​GTG​AGG​GAG​CGA​T-3′, reverse: 5′-TGA​GCC​AAA​GGG​ATC​TAC​GC-3′; *AL451069.3*, forward: 5′-GGG​ACA​CGG​ACC​TAG​ACA​CT-3′, reverse: 5′-CCT​GCA​AGA​CCG​TAG​CCT​C-3′; *ALO31985.3*, forward: 5′-TCT​CAC​TAT​GTT​GCT​GGA​CTG​G-3′, reverse: 5′-CCA​CAG​ATC​ACT​AAC​ACG​CC-3′. We used glyceraldehyde-3-phosphate dehydrogenase (GAPDH) as the internal reference, and the data were analyzed using the 2^–ΔΔCt^ approach. The expression levels of the three lncRNAs were compared using an unpaired t-test.

## 3 Results

### 3.1 Defining senescence-related lncRNAs

**FIGURE 1:**
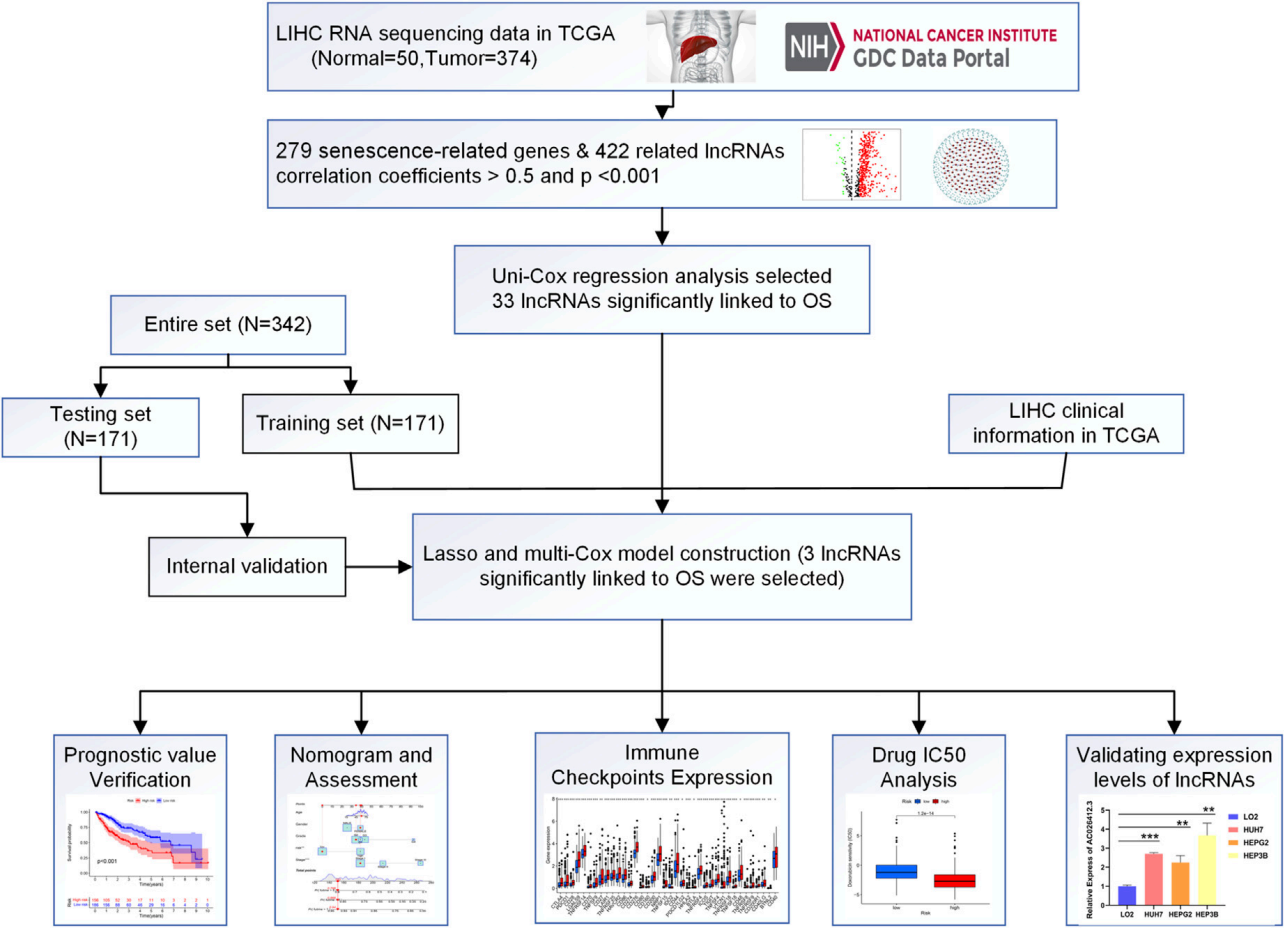
Flow diagram of the study (LIHC: Liver hepatocellular carcinoma; N: Number; OS: Overall suvival).

**FIGURE 2:**
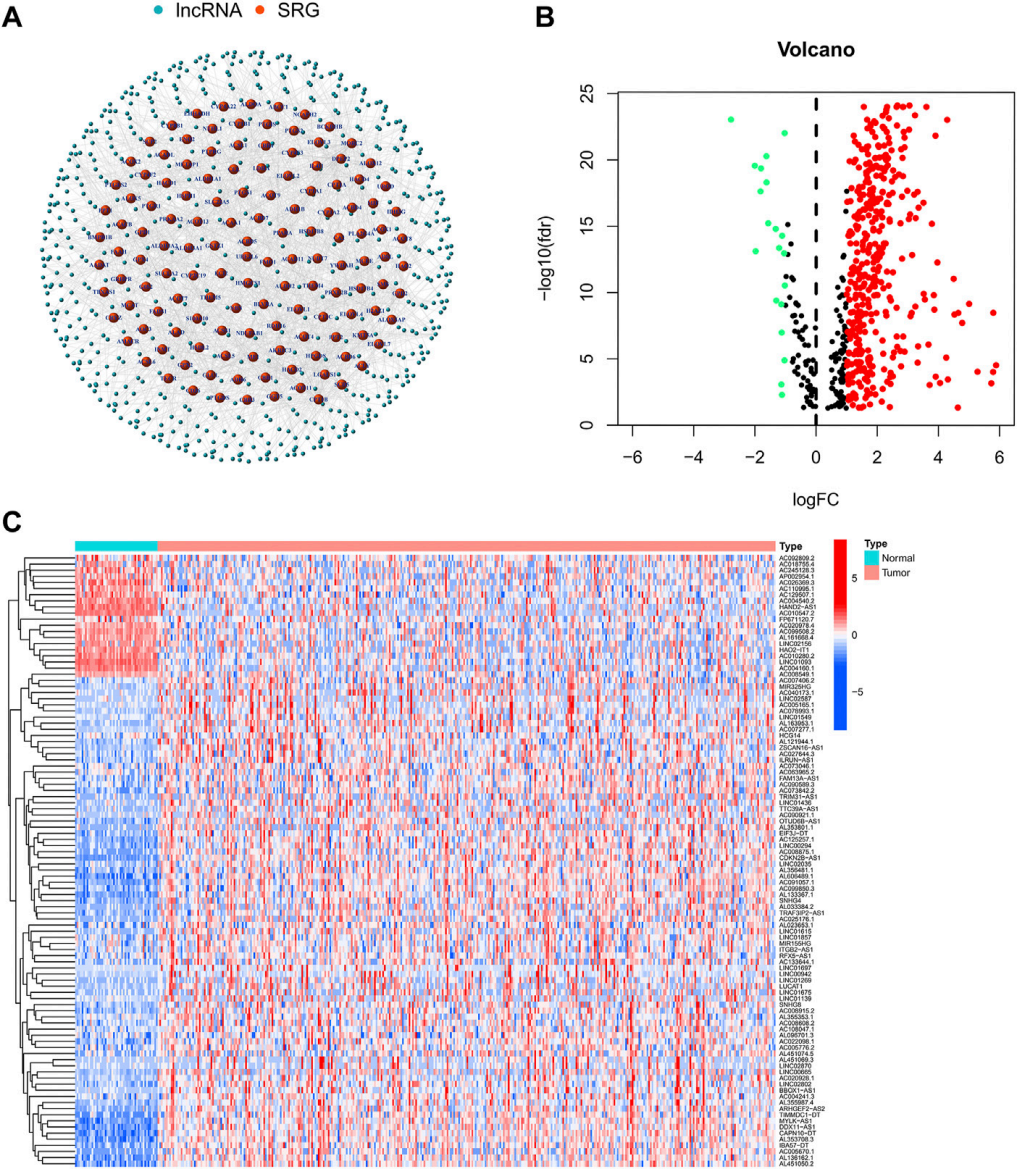
**(A)**The network showing the correlation between DEsrlncRNAs and mRNAs and **(B,C)** the heatmap and volcano plots of DEsrlncRNAs from the TCGA dataset.

The flow-diagram of our study is shown in Figure 1. We downloaded 50 normal samples and 374 tumor samples from the TCGA database to identify the srlnRNAs. Next, 422 srlncRNAs (Figure 2A) were obtained by using the co-expression analysis of 279 senescence-related genes and DElncRNAs between normal and tumor samples. Of these, 402 were up-regulated (Figures 2B, C).

### 3.2 Establishment and validation of the model

**FIGURE 3:**
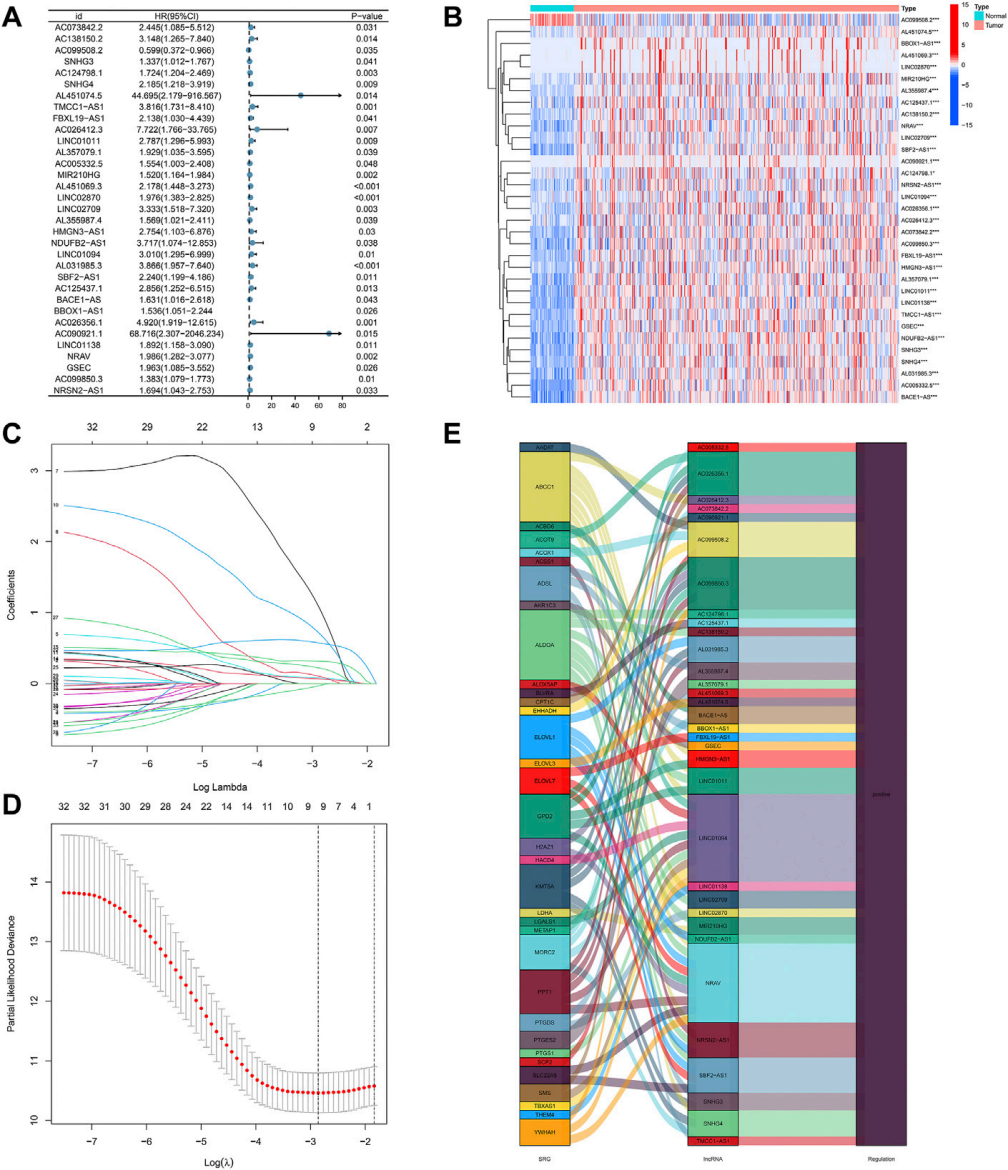
**(A)** 33 lncRNAs extracted by using the uni-Cox regression analysis, **(B)** the heat map of 33 prognostic lncRNAs, **(C,D)** senescence-related lncRNAs screened by the Lasso regression analysis, and **(E)** the Sankey diagram of 33 senescence genes and related lncRNAs.

Using the univariate-Cox regression analysis (Figure 3A), 33 srlncRNAs that significantly associated with the overall survival were identified and are displayed in a heatmap (Figure 3B). LASSO and multi-Cox regression analyses were used to further screen these lncRNAs, and three lncRNAs related to senescence were extracted in HCC (Figures 3C, D). In addition, all lncRNAs were up-regulated in the Sankey diagram (Figure 3E).

The risk score was calculated using the following formula: risk score = *AC026412.3* × (1.6474) + *AL451069.3* × (0.6620) + *AL031985.3* × (1.0340).

**FIGURE 4:**
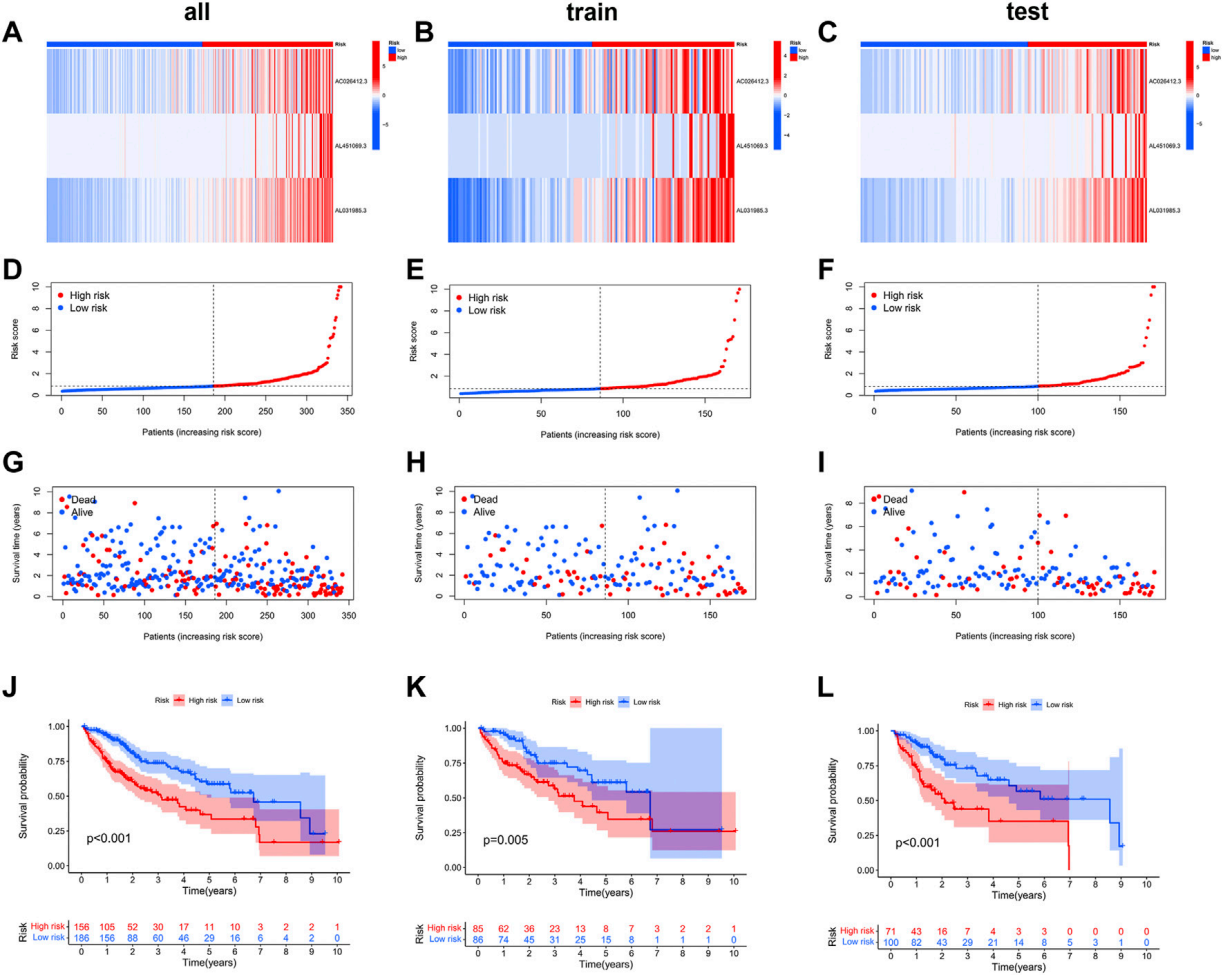
The heat map **(A–C)**, risk score **(D–L)**, survival status **(G–I),** and Kaplan–Meier curves **(J–L)** of the two groups in the training, testing, and entire sets, respectively.

We then compared the distribution of the risk scores, survival status, survival time, and associated expression criteria of these lncRNAs for the low- and high-risk groups in the training, testing, and entire sets. These results suggested that the high-risk group had a poorer prognosis (Figures 4A–L).

**FIGURE 5:**
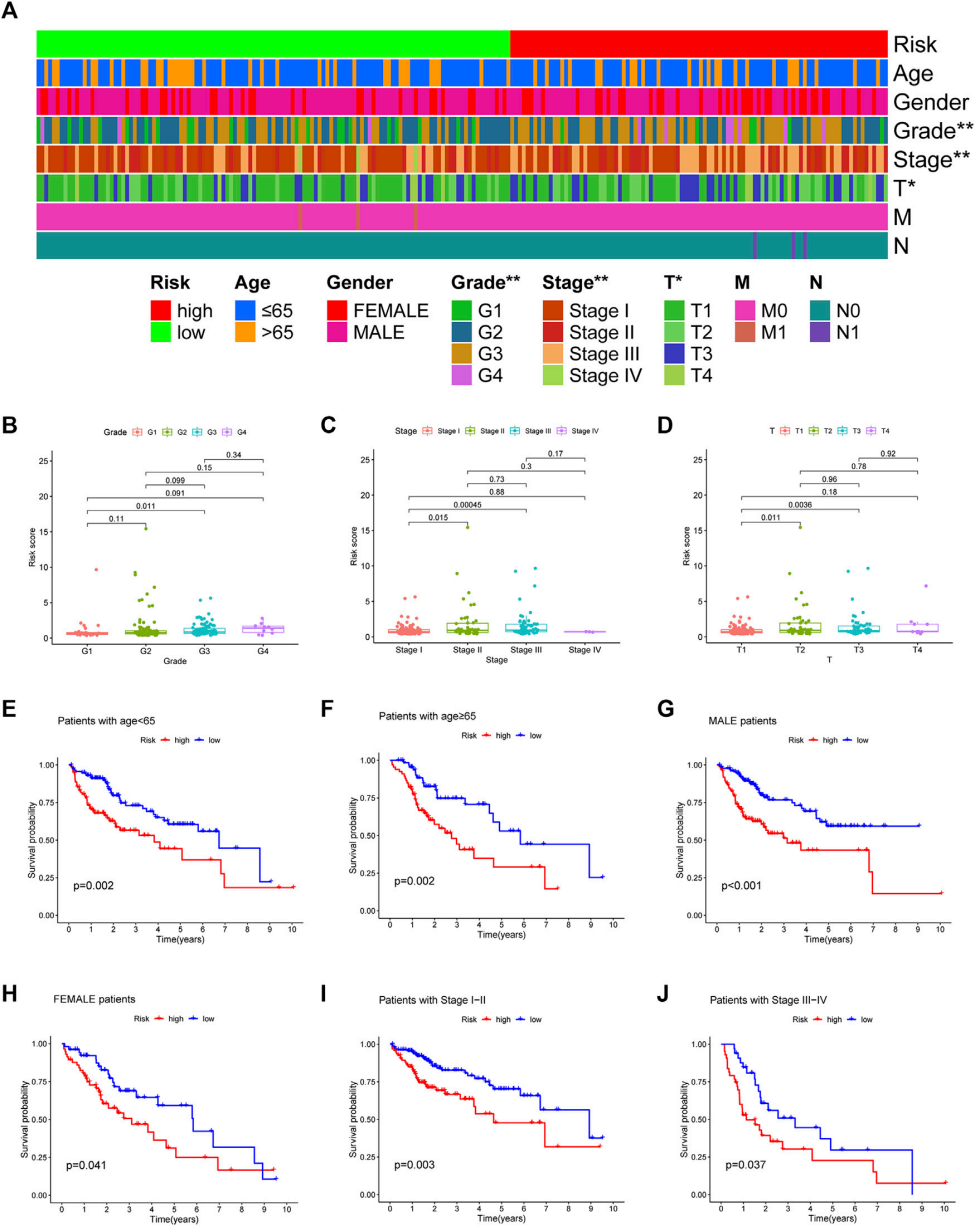
The strip chart **(A)** and Scatter diagram **(B–D)** showing significant correlation of the tumor grade, American Joint Committee on Cancer stage, and T stage with risk scores. *p < 0.05 and **p < 0.01. **(E–J)** The Kaplan–Meier analysis showing a longer survival time in low-risk group patients.

According to chi-square tests (Figure 5A) and Wilcoxon signed-rank test, the risk score was significantly associated with the clinical grade (Figure 5B), American Joint Committee on Cancer stage (Figure 5C), and T stage (Figure 5D). In addition, conventional clinicopathological characteristics, including age, sex, and stage, also showed that the high-risk group had worse prognoses (Figures 5E–J). These results indicate that the risk model is highly consistent with the American Joint Committee on Cancer staging system and has a better ability to predict prognosis.

**FIGURE 6:**
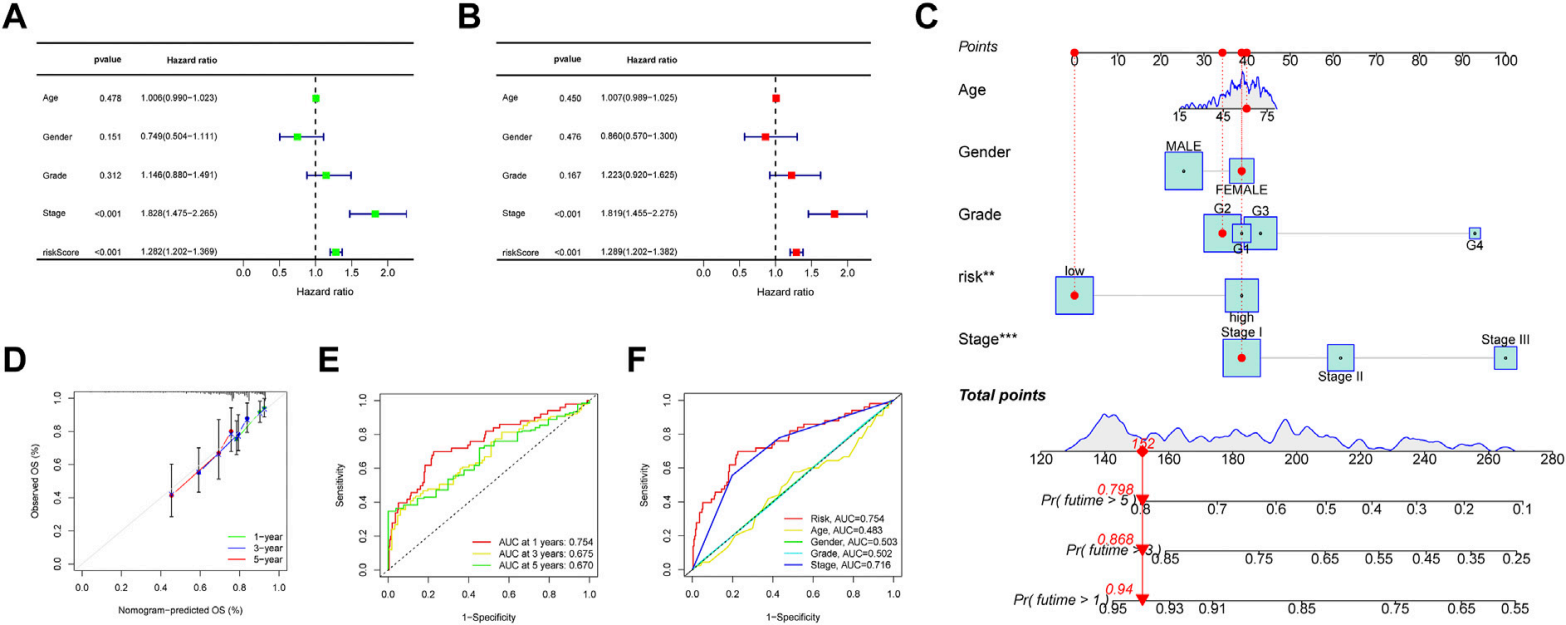
**(A, B)** Forest plots of the uni- and multi-Cox regression analyses in HCC, **(C)** the nomogram-combined risk score, age, and tumor stage to predict the 1-, 3-, and 5-year OS in HCC and **(D)** evaluation of the nomogram by correlating it with the calibration curves. **(E)** The ROC curves of the model for prognosis, and **(F)** the ROC curves of the risk score and clinicopathologic features.

Prognostic factors were detected in the uni- and multi-Cox regression analyses (Figures 6A,B) and a nomogram was constructed using the risk scores and other clinical characteristics to better predict the survival of patients with HCC (Figure 6C). What’s more, the nomogram correlated with the actual observations, as shown in the calibration curve (Figure 6D). The 1-, 3-, and 5-year areas under the ROC curve of the entire set were 0.754, 0.675, and 0.670, respectively (Figure 6E). Compared to other clinicopathological features, the risk score had the largest area under the ROC curve (Figure 6F).

#### 3.2.1 Gene set enrichment analysis

**FIGURE 7:**
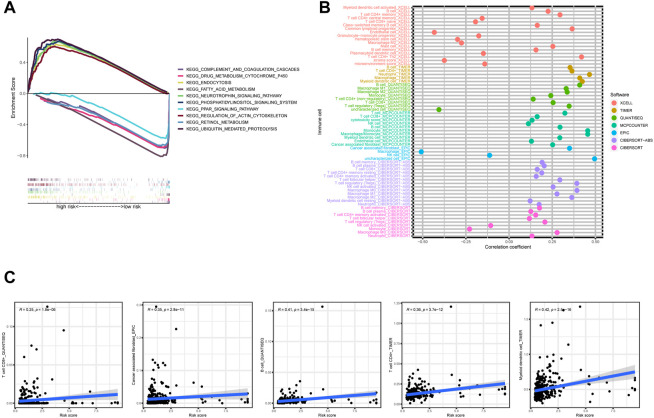
**(A)** The top five pathways with enrichment in the high- and low-risk groups with the GSEA analysis, **(B)** the bubble chart showing risk groups and immune cells, and **(C)** line graph demonstrating risk score and immune cells.

To explore the different biological functions in the two risk groups, the GSEA software was used to identify the top five pathways in the two risk groups with the criteria of false discovery rate < 0.25, |NES| >1.5, and *p* < 0.05. In fact, most of the pathways were associated with tumorigenesis or immunity, such as the “fatty acid metabolism”, “peroxisome proliferator-activated receptors signaling pathway”, and “complement and coagulation cascades” (Figure 7A). Therefore, we performed an immunity analysis of the model.

### 3.3 The exploration of the risk model for immunotherapy

**FIGURE 8:**
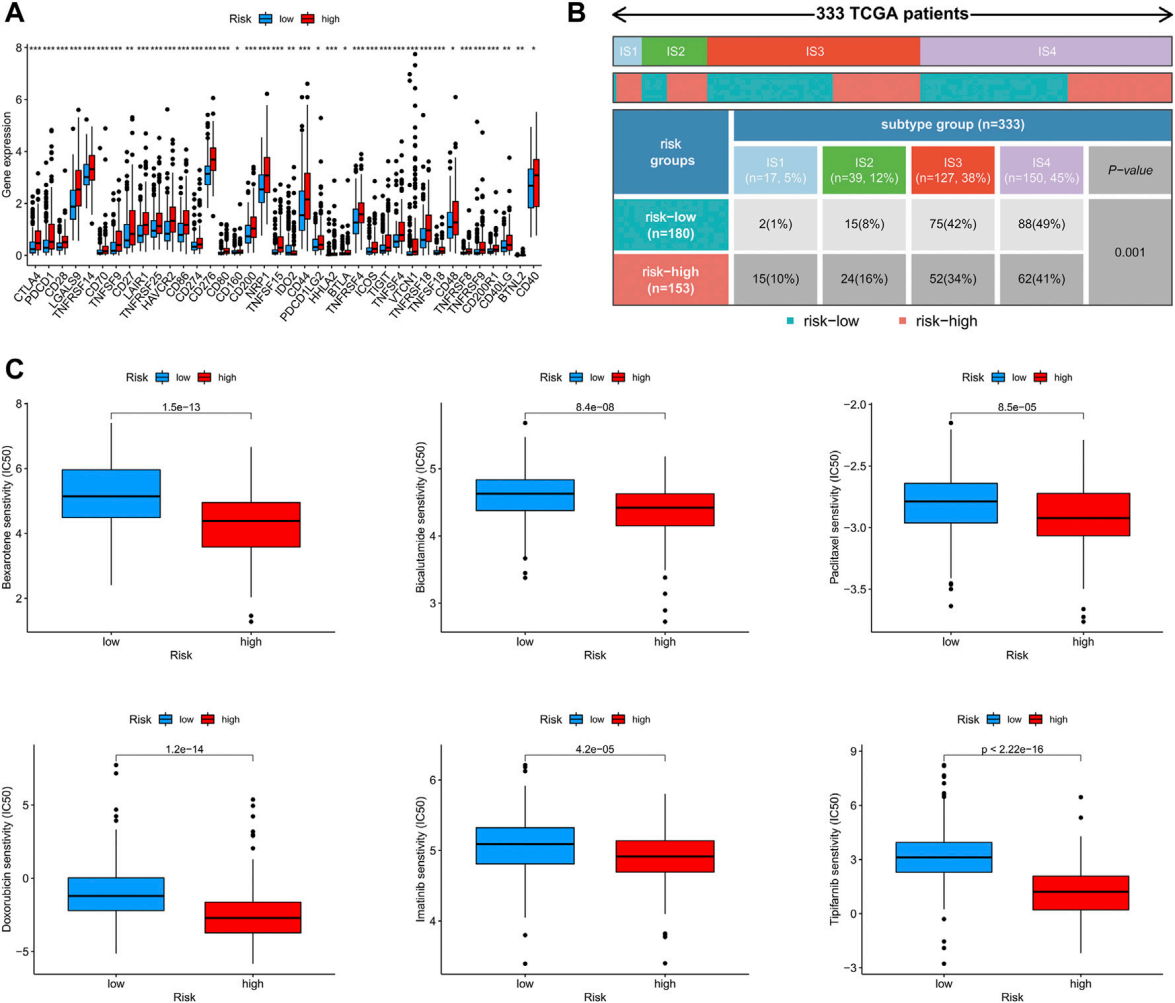
**(A)** The diﬀerence of 38 checkpoint expressions in the risk groups, **(B)** the immune subtype of high- and low-risk groups, and **(C)** the immunotherapy prediction of the risk groups.

Using Spearman’s correlation and Wilcoxon signed-rank tests, the risk score was found to be related to several widely studied immune cells (such as B cells, CD8^+^ T cells, and cancer-associated fibroblasts) on different platforms (Figures 7B,C). The expression of the immune checkpoint-related genes was higher in the high-risk group than in the low-risk group (Figure 8A). This implies that patients in the high-risk group could select checkpoint inhibitors that are more appropriate for immunotherapy (Kono et al., 2020). Moreover, the high-risk group had a larger proportion of immune subtypes (IS) 1 and 2 in the immunity landscape and a smaller proportion of 3 (Figure 8B), which means that it had a poorer prognosis (the immune landscape of cancer). Consistent with previous reports, there were more chemotherapeutics with lower IC50 values in the high-risk group (Figure 8C), such as paclitaxel (Liu et al., 2020) (Figure 8C).

### 3.4 Validating the expression levels of *AC026412.3*, *AL451069.3*, and *AL031985.3*

**FIGURE 9:**
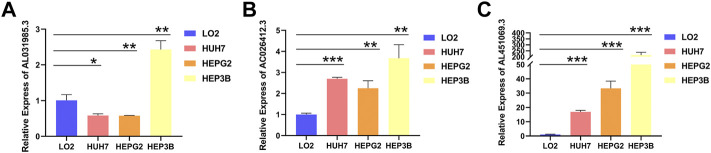
The relative RNA level of AL031985.3 **(A)**, AC026412.3 **(B)**, and AL451069.3 **(C)** in normal hepatic and hepatoma cell lines. Data is presented as Mean with SD, *p < 0.05, **p < 0.01, ***p < 0.001.

To explore the expression levels of *AC026412.3*, *AL451069.3*, and *AL031985.3*, qPCR was performed to test the normal hepatic and hepatoma cell lines. The expression levels of *AL451069.3* and *AC026412.3* in hepatoma cell lines were much higher than those in a normal hepatic cell line (Figure 9). Furthermore, the expression levels of these srlncRNAs were different in diverse hepatoma cell lines (Supplementary Figure S1).

## 4 Discussion

### 4.1 Resource identification initiative

Cellular senescence has been found to play a role in the development and progression of various types of malignant tumors, including HCC, and is considered a barrier to the progression from a chronic liver disease to HCC. Xiang et al. (2021) reported that the lncRNA PINT87aa was upregulated in senescent HCC cells and could induce cell cycle arrest by blocking FOXM1-mediated PHB2. Mittermeier et al. (2020) described the characteristics and functions of cellular senescence in the development of novel drug targets for HCC therapies. Karakousis et al. (2020) suggested that hepatitis B is a link between cellular senescence and HCC development. A better understanding of the role of cellular senescence in HCC may provide a new perspective for HCC treatment and aid in the development of new therapeutic methods.

The expression patterns and clinical information of 377 patients with HCC were downloaded from the TCGA database, senescence-related genes were identified from the CellAge public database, and a co-expression analysis was performed to identify the genes potentially involved in HCC. Three prognosis-related DEsrlncRNAs were screened to construct a signature using LASSO and uni-Cox regression analyses: *AC026412.3*, *AL451069.3*, and *AL031985.3*. Among the three srlncRNAs, *AL031985.3* has been identified as a potential therapeutic target in HCC in a previous study (Jia et al., 2020). Moreover, the Sankey diagram showed that the three srlncRNAs were associated with a few coding genes, including *PPT1*, *PTGDS*, and *ELOVL1*. High *PPT1* expression is associated with poor prognosis in patients with HCC, and PPT1 inhibition could enhance the sensitivity to sorafenib therapy in HCC (Xu et al., 2022). *PTGDs* are prognostic biomarkers of breast cancer (Adekeye et al., 2022). Hama et al. (2021) demonstrated that the expression of *ELOVL1* was significantly higher in CRC tissues than in normal tissues. These results suggest that the three identified srlncRNAs may serve as potential biomarkers for cancer diagnosis and treatment.

The risk score was calculated based on the expression levels of the three srlncRNAs, and patients in each cohort were separated into high- and low-risk groups according to the calculated risk score. The Kaplan–Meier curve showed that patients with a low risk score had a better prognosis. Based on the results of the uni- and multi-Cox regression analyses, the risk score could be an independent prognostic factor for patients with HCC. In addition, nomograms are widely used as tools in oncology, particularly for survival prediction (Iasonos et al., 2008; Balachandran et al., 2015). The nomogram model and calibration plot showed good prediction efficiency for HCC prognosis. Moreover, the correlation between risk scores and clinical features of HCC was also analyzed; the risk score was significantly related to the tumor grade, AJCC stage, and T stage, indicating that the risk score can be used for predicting the occurence and development of HCC. However, the results of the Wilcoxon signed-rank test showed that the advancing stages (G4, stage IV, and T4) were not significantly related to the calculated risk score. Because the sample content of the TCGA database is too small, we will have to collect more samples to re-validate.

Based on the results of GSEA, we focused our attention on the immunity factors. Previous research has suggested that tumor-infiltrating CD4^+^ T cells can upregulate the immune checkpoint genes (Toor et al., 2019). We used TIMER2.0 to assess the relationship between the risk score and tumor-infiltrating immune cells (Van Veldhoven et al., 2011; Newman et al., 2015; Becht et al., 2016; Aran et al., 2017; Li et al., 2017; Finotello et al., 2019; Tamminga et al., 2020). The results revealed that the risk score was positively related to B cells, CD8^+^ T cells, and cancer-associated fibroblasts. To further explore the potential of checkpoint blockade therapy and chemotherapy, we compared the two groups’ expression levels of the immune checkpoint genes and found 38 checkpoint genes that were differentially expressed between the two groups in this study. Consistent with the alteration of the checkpoint genes, the IC50 values of six common chemotherapeutics were higher in the low-risk group. These findings suggest that patients with high-risk scores may be more suitable for immunotherapy and chemotherapy.

Thorsson et al. (2018) identified the ISs, including wound healing (IS1), IFN-γ dominant (IS2), inflammatory (IS3), lymphocyte-depleted (IS4), immunologically quiet (IS5), and TGF-β dominant (IS6) types of cancer. It was observed that IS1 and IS2 had worse outcomes, IS3 had a favorable prognosis, and IS3 was enriched in PBRM1 mutation. Moreover, patients with PBRM1 mutations were more responsive to immunotherapy (Miao et al., 2018). Our study indicated that patients with low-risk scores had a larger proportion of IS3, which comports with the Kaplan–Meier curve.

However, our study has few limitations. First, our analysis was based on public datasets and retrospectively collected samples, which may have caused an inherent case selection bias. Second, further experiments are required to confirm our findings. Finally, clinical features related to surgery, neoadjuvant chemotherapy, and tumor markers were not included in our study, and clinical cases are required to further validate our conclusions.

In conclusion, the cellular senescence-based prognostic signature constructed in this study may be useful for predicting the survival and guiding clinical therapies for HCC. Our findings may improve the understanding of cellular senescence in HCC and provide more effective treatment strategies. However, additional experiments and clinical cases are required to validate these findings.

## Data Availability

The datasets presented in this study can be found in online repositories. The names of the repository/repositories and accession number(s) can be found in the article/Supplementary Material.

## Supplementary material

Supplementary Table S1 279 senescence-related genes.

Supplementary Table S2 422 senescence-related lncRNAs.

Supplementary Table S3 33 lncRNAs extracted by univariate Cox regression analysis.

Supplementary Table S4 3 lncRNAs used to construct prognostic model.

Supplementary Table S5 Raw data of qRT-PCR.

Supplementary Table S6 Data and scripts used in the study are uploaded to Jianguoyun, and the links are included.

## Abbreviations

DElncRNAs, differentially expressed lncRNAs; GAPDH, glyceraldehyde-3-phosphate dehydrogenase; GSEA, Gene set enrichment analysis; HCC, Hepatocellular carcinoma; IRGPI, immune-related gene prognostic index; IS, immune subtypes; KM, Kaplan-Meier; LASSO, least absolute shrinkage and selection operator; lncRNA, long non-coding ribonucleic acids; multi-Cox, multivariate Cox; IC50, Half-maximal inhibitory concentration; qPCR, quantitative real-time PCR; ROC, receiver operating characteristic; SRGs, senescence-related genes; srlncRNAs, senescence-related lncRNAs; TCGA, The Cancer Genome Atlas; uni-Cox, univariate Cox.
