# Supplementary material for: Development and validation of a novel cellular senescence-related prognostic signature for predicting the survival and immune landscape in hepatocellular carcinoma
Source: Front Genet. 2022 Sep 6;13:949110. doi: 10.3389/fgene.2022.949110 (PMC9485671; doi:10.3389/fgene.2022.949110)
Supplement: Supplementary file 6 [file Table6.DOCX]

https://www.jianguoyun.com/p/DcCDYYEQ4tzMChj3uMIEIAA
